# 245. Using Probability of Community-Acquired Pneumonia to Tailor Antimicrobials Among Inpatients (UP-CAPTAIN): A Pragmatic Randomized Trial Evaluating the Impact of Guided Test Interpretation on Antibiotic Use among Patients with Suspected Community-Acquired Respiratory Infection

**DOI:** 10.1093/ofid/ofaf695.088

**Published:** 2026-01-11

**Authors:** Jonathan Baghdadi, Anthony Harris, Lisa Pineles, Shatha AlShanqeeti, Danica Palacio, Drew W Charles, Emily L Heil, Kimberly C Claeys, J A C Q U E L I N E T BORK, Melinda M Neuhauser, Sarah Kabbani, Sarah Sommerkamp, R Gentry Wilkerson, Mark Sutherland, J Kristie Johnson, Daniel J Morgan

**Affiliations:** University of Maryland School of Medicine, Baltimore, Maryland; University of Maryland School of Medicine, Baltimore, Maryland; University of Maryland School of Medicine, Baltimore, Maryland; University of Maryland, Baltimore, MD; Perelman School of Medicine, University of Pennsylvania, Philadelphia, Pennsylvania; Medical University of South Carolina, Charleston, South Carolina; University of Maryland School of Pharmacy, Baltimore, MD; University of Maryland Baltimore, Baltimore, Maryland; University of Maryland School of Medicine, Baltimore, Maryland; Division of Healthcare Quality Promotion, Centers for Disease Control and Prevention,, Atlanta, GA; Centers for Disease Control and Prevention, Atlanta, GA; University of Maryland School of Medicine, Baltimore, Maryland; University of Maryland School of Medicine, Baltimore, Maryland; University of Maryland School of Medicine, Baltimore, Maryland; University of Maryland School of Medicine, Baltimore, Maryland; University of Maryland School of Medicine, Baltimore, Maryland

## Abstract

**Background:**

Respiratory illness is the most common reason for unnecessary antibiotic therapy in hospitalized adults. Although procalcitonin and respiratory virus testing help differentiate between viral and bacterial illness, they tend not to influence antibiotic decision-making.

**Figure 1.**
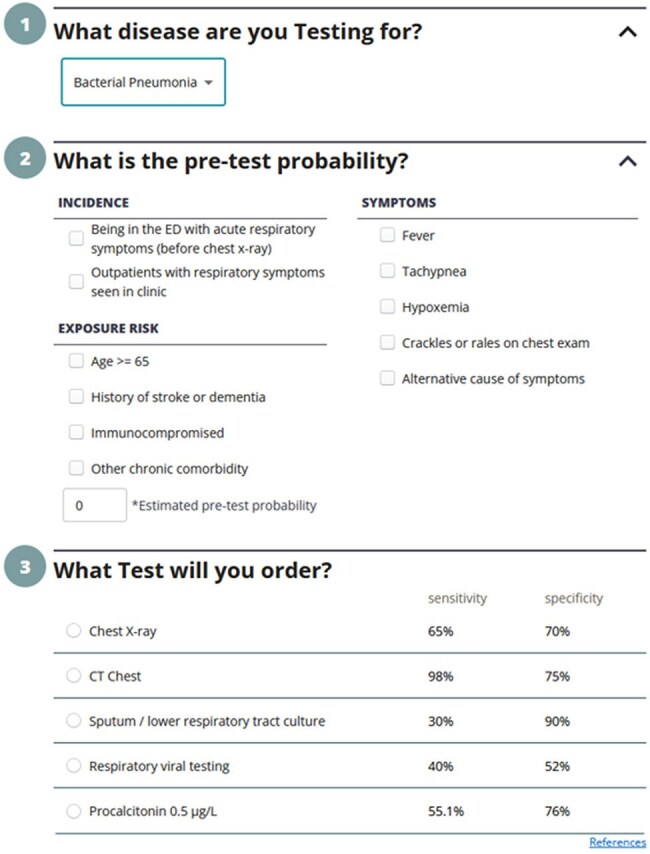
Testing Wisely Disease Risk Calculator for Bacterial Pneumonia

**Figure 2.**
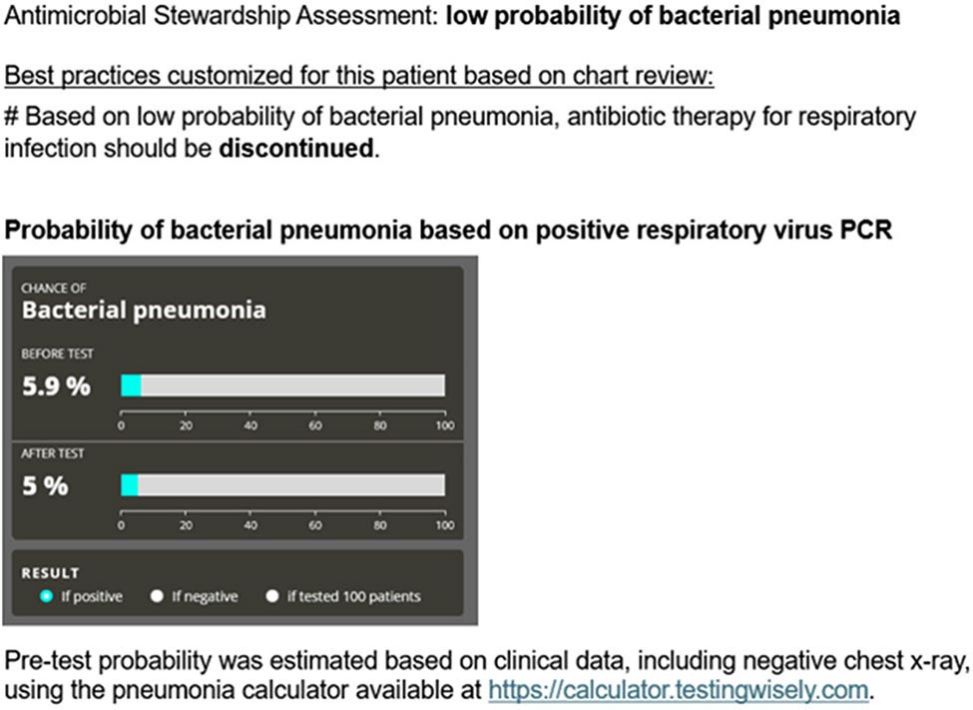
Example of Templated Note for a Patient with Low Post-test Probability of Bacterial Pneumonia

**Methods:**

We conducted a pragmatic randomized-controlled trial comparing antimicrobial stewardship-guided test interpretation versus usual care among hospitalized adults receiving antibiotics for suspected respiratory infection with either low serum procalcitonin or positive respiratory virus testing at 2 hospitals (NCT05976581). The intervention involved chart review to estimate post-test probability of bacterial pneumonia based on either the low procalcitonin or positive respiratory viral panel and available imaging using an online calculator (Figure 1). Post-test probability was then documented in a note in the patient’s chart along with templated recommendations (Figure 2). When post-test probability was low, antibiotics were recommended to be discontinued. When post-test probability was medium or high, recommendations focused on tailoring antibiotics and setting an appropriate duration of therapy. An electronic message referring to the note and summarizing recommendations was also sent to the clinical team.

**Figure:**
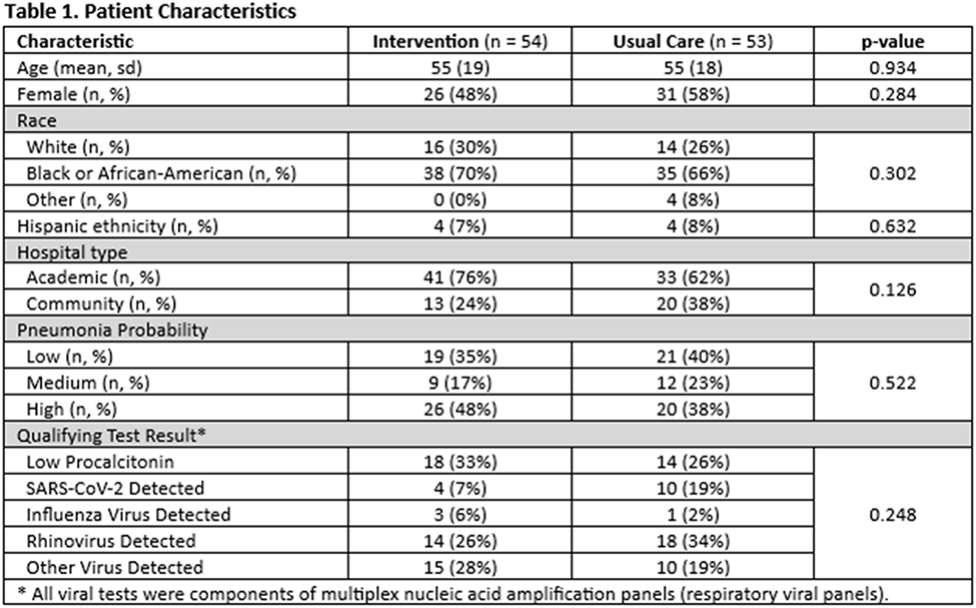

**Figure:**
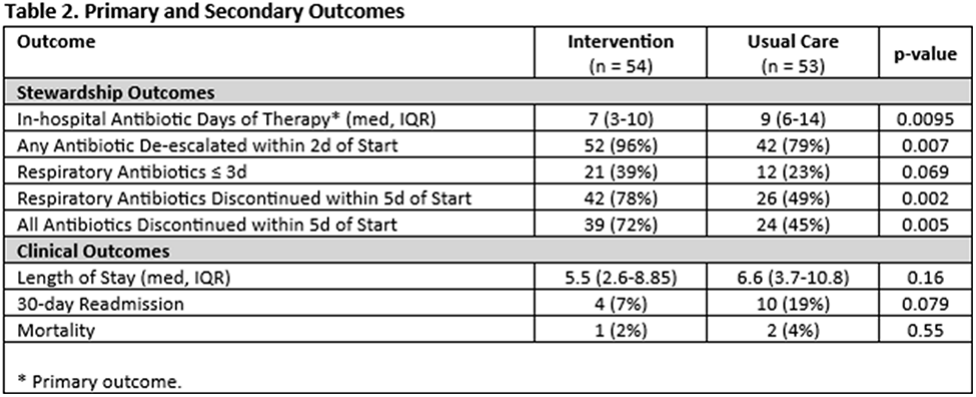

**Results:**

Between 11/1/2023 and 1/10/2025, 107 adult inpatients were randomized, including 54 to the intervention and 53 to usual care (Table 1). Thirty percent of enrolled patients had a low serum procalcitonin, and 70% had a positive respiratory virus panel. The intervention significantly decreased antibiotic use (median hospital days of antibiotic therapy 7.5 versus 11, p = 0.005, Table 2) and increased discontinuation of all respiratory antibiotics within 5 days of initiation (76% versus 49%, p = 0.004). Clinical outcomes, including length of stay (5.5d with intervention versus 6.6d with usual care, p = 0.16) and 30-day readmission (7% with intervention versus 19% with usual care, p = 0.079), were equivalent between groups.

**Conclusion:**

Guided interpretation of standard diagnostic tests for viral infection substantially reduced unnecessary antibiotic use for hospitalized adults with community-acquired respiratory illness.

**Disclosures:**

Anthony Harris, MD, MPH, UpToDate Wolters Kluwer Health: Infection control section editor Emily L. Heil, PharmD, MS, Wolters-Kluwer: Advisor/Consultant

